# Preparing Hydrophobic Cellulose Nanofibers-SiO_2_ Films and Coating by One-Step Mechanochemical Method

**DOI:** 10.3390/polym14204413

**Published:** 2022-10-19

**Authors:** Xi Chen, Lijiaqi Zhang, Min Wu, Yong Huang

**Affiliations:** 1 National Engineering Research Center of Engineering and Eco-Plastics, Technical Institute of Physics and Chemistry, Chinese Academy of Sciences, Beijing 100190, China; 2 College of Materials Science and Opto-Electronic Technology, University of Chinese Academy of Sciences, Beijing 100049, China

**Keywords:** cellulose nanofiber, hydrophobic SiO_2_ nanoparticle, composite, hydrophobic coating, optical tunable film

## Abstract

Green and sustainable cellulose-based hydrophobic coatings are increasingly the subject of scientific and industrial research. However, few researchers pay attention to preparing it by a one-step method. Therefore, a superhydrophobic coating composed of hydrophobic SiO_2_ and cellulose nanofiber modified by 3,4-dichlorophenyl isocyanate was manufactured through one-step ball milling. It was found that the ball milling can promote SiO_2_ dispersion and achieve the preparation of modified nanocellulose, which further disperse SiO_2_ nanoparticles to form film or coating. Compared with the ultrasonic dispersion method, the composite coating prepared by ball milling method can obtain higher water contact angle and more stable hydrophobic properties. The hydrophobic cellulose nanofiber can load 1.5 equivalents of SiO_2_ nanoparticles to form a uniform film with the water contact angle of 158.0° and low moisture absorption. When this nanocomposite is used as a coating material, it can impart super-hydrophobicity to paper surface with water contact angle of 155.8°. This work provides a facile way to prepare superhydrophobic nanocellulose/nanoparticles composite coatings and films, thereby broadening the ways of dispersing nanoparticles and constructing superhydrophobic coatings.

## 1. Introduction

Superhydrophobic surfaces have fascinated the interest of industry and researchers because of their versatility and potential application values [1,2,3,4]. To construct a superhydrophobic surface, the roughness structure and low-surface-energy should be achieved simultaneously [5,6,7,8]. Compared with etching rough structure, it is a low-cost and efficient strategy to prepare the size agent with hydrophobic nanoparticles to achieve a hierarchical microstructure for the underlying substrates [9,10]. Among many hydrophobic nanoparticles, SiO_2_ nanoparticles with distinctive colloidal stability is promising candidates for the preparation of superhydrophobic coatings [11,12]. Common superhydrophobic coatings are often prepared by compounding SiO_2_ nanoparticles with polymer materials, such as hybrid polyvinylidene fluoride [13], fluoroacrylate block copolymers [14], polystyrene [15,16], PDMS [17,18], and cellulose [19,20,21]. Utilization of degradable cellulose nanofiber (CNF) as a reaction medium to control the wettability of the substrate is in conformity with economic and environmental development but challenging due to the hydrophilicity of cellulose [22]. Therefore, the CNF in the coating often needs to be hydrophobically modified. It has been reported that when hydrophobic coatings are prepared by mixing CNF and SiO_2_, the hydrophobicity is usually modified after in situ generation of nanoparticles on the surface of nanofibers by a sol-gel method [20,23]. Alternatively, hydrophobic CNF and nanoparticles are prepared separately, and then further mixed to obtain hydrophobic coatings [24]. Nevertheless, few researchers focus on dispersing SiO_2_ nanoparticles while defibrillating and hydrophobizing cellulose.

The performance advantages of nanocomposites coating depend on the good dispersion of the nanoparticles [25]. Nanoparticles are often dispersed by surfactants or ultra-high shear forces [16,26,27,28]. Studies in multiple fields demonstrate that it is competent to manufacture CNF-based superhydrophobic coatings, because CNF is validated as a dispersant or surfactant substitute to disperse nanomaterials [29,30,31,32]. Moreover, the inherent structural characteristics of CNF are also conducive to reconstructing micro/nano scale rough structures, thereby preparing super-hydrophobic coatings [10,33]. In the previous work, we demonstrated that the hydrophobic SiO_2_ nanoparticles can be dispersed by CNF to reduce agglomeration [19]. However, few studies have been conducted to obtain hydrophobic nanoparticles/nanocellulose composite coatings by the one-step method. Ball milling, thanks to its unique high shear action, can defibrillate and hydrophobically modify cellulose at the same time [34,35]. Moreover, we found that the CNF modified with 3,4-dichlorobenzene isocyanate has good adhesion and film-forming properties. Based on the purpose of the one-step preparation of superhydrophobic coatings, we believe that ball milling is an effective strategy for the nanomatization of cellulose while dispersing hydrophobic SiO_2_ nanoparticles. It is speculated that the defibrination of cellulose will further promote the dispersion of nanoparticles when cellulose and nanoparticles are co-ball-milled. Therefore, the SiO_2_ nanoparticles mixed in cellulose will redisperse on the surface of cellulose nanofiber to construct superhydrophobic coatings.

In this work, the hydrophobic CNF/SiO_2_ nanoparticles composite coatings were prepared by the one-step ball milling method, which was applied on hydrophobic films and paper. The modifier of cellulose is 3,4-dichlorobenzene isocyanate. The influence of two high-shear methods (ultrasonic and ball milling) on the dispersion of hydrophobic SiO_2_ nanoparticles and the effect of SiO_2_ content on the wettability of the coating were discussed. This study may provide an effective and facile way to construct a superhydrophobic coating with micro/nano rough structure.

## 2. Materials and Methods

### 2.1. Materials

Bleached softwood pulp (*M*_η_ = 3.8 × 10^5^ g/mol) was provided by Zhejiang (China) Jinchang Paper Co., Ltd. 3,4-dichlorophenyl isocyanate (DCPI, 97%) was purchased from Aladdin, China. Hydrophobic SiO_2_ nanoparticles (HN-SiO_2_) was purchased from EVONIK-DEUSSA, with the average particle size of 14 nm. Dimethyl sulfoxide (DMSO, AR), N, N-dimethylformamide (DMF, AR) were purchased from Sinopharm Chemical Reagent Co., Ltd., Shanghai, China. All reagents and materials were used without further purification.

### 2.2. Preparation of Hydrophobic CNF/HN-SiO_2_ Suspension

The HN-SiO_2_ nanoparticles were ball milled with 0.50 g of bleached softwood pulp powder and 1.74 g of DCPI in 20 mL of DMSO for 6 h to obtain the CNF/HN-SiO_2_ complex. The weight ratio of HN-SiO_2_ to cellulose was 0.1, 0.25, 0.5, 0.75, 1, 1.5. The product was washed with DMF by centrifugation and denoted as HS_n_D_6_, where *n* represents the weight percentage of HN-SiO_2_ to D_6_-CNF.

For comparison, 0.50 g of softwood pulp powder was mixed with 1.74 g of DCPI for 6 h, which was named as D_6_-CNF. Then, referring to the weight ratio in HS_n_D_6_, D_6_-CNF and HN-SiO_2_ were mixed by ultrasonic dispersion method directly, which coded as D_6_ + HS_n_. Without special instructions, HS_n_D_6_ and D_6_ + HS_n_ are collectively referred to as CNF/HN-SiO_2_.

### 2.3. Preparation of CNF/HN-SiO_2_ Films

First, 2 mL of 0.5 wt% CNF/HN-SiO_2_ dispersions were evenly dropped on a 24 × 40 mm^2^ glass slide, and dried at 60 °C to form the film with a thickness of 15 μm. The solvent was completely removed by vacuum drying at 100 °C for 30 min.

### 2.4. Preparation of HS_n_D_6_ Coated Paper

HS_n_D_6_ dispersion was coated on kraft paper surface by wire rod coater to form a hydrophobic coating surface with 6 g/m^2^ sizing amount and dried at 100 °C for 5 min.

### 2.5. Characterization

The functional groups of CNF/HN-SiO_2_ films were analyzed by ATR mode of infrared spectrophotometer (FTIR, Excalibur 3100). The scanning range was 400–4000 cm^−1^, the resolution was 4 cm^−1^, and the cycle was 64 times.

The content of HN-SiO_2_ in HS_n_D_6_ was analyzed by thermogravimetric analyzer (TGA, PE Pyrisl). The gas flow rate was 25 mL/min, the sample mass was 5–10 mg. The specimens were tested with a heating rate within 10 °C/min in the range of 30–600 °C under oxygen atmosphere.

The morphology of CNF in HS_n_D_6_ was observed by atomic force microscopy (AFM, Bruker Multimode 8). The suspension was dispersed in DMF and sonicated for 3 min with the concentration of 5 ppm, then 4 μL of the dispersion was deposited on a freshly cleaved mica plate and dried at room temperature. The roughness of the CNF/HN-SiO_2_ films was tested by AFM. The morphology of the composite film and hydrophobically modified paper was observed by scanning electron microscopy (SEM, JEOL JSM-4800).

The water contact angle (WCA) on the coating surface was measured at ambient temperature by an OCA 20 contact angle system. The contact angle was measured after the water drop remained on the interface for 5 s. For WCA measurement, 3 μL of deionized water was dropped onto the CNF/HN-SiO_2_ films and its coating surface and at least three measurements were averaged.

The water absorption capacity was evaluated by immersing the film into water and wiping the film surface with dried filter paper to remove excess water. Then, the film was weighed and the water absorption capacity was calculated by following Equation (1), where *m*_0_ (g) and *m_x_* (g) are the weight of the film before and after absorption, and *x* (h) is the soaking time.

(1) $$Water\;absorption\left(\%\right)=(m_{x}-m_{0})/m_{0}\times 100$$

The total transparency (*T_total_*) of the film was measured by UV-Vis-NIR spectrometer (Varian Cary 7000) equipped with an integrating sphere component. The direct transmittance (*T_direct_*) was measured by UV−-Vis-NIR spectrometer measurements (Varian Cary 5000). The optical haze was calculated by Equation (2).

(2) $$Haze\left(\%\right)=(T_{total}-T_{direct})/T_{total}\times 100$$

## 3. Results and Discussion

**Figure 1:**
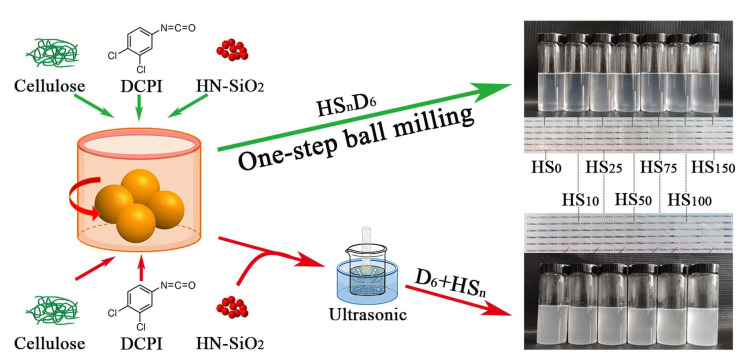
Schematic of the preparation of hydrophobic CNF/HN-SiO_2_ composite by ball milling.

The schematic of the preparation of CNF/HN-SiO_2_ composite suspensions and films by means of CNF-assisted dispersion of hydrophobic SiO_2_ nanoparticles by ultrasonic and ball milling is shown in Figure 1. According to previous work, isocyanate groups of DCPI and hydroxyl groups on cellulose surface can undergo a urethane reaction to form hydrophobic chains. Due to the adhesion of the modified D_6_-CNF, the added HN-SiO_2_ nanoparticles can be adhered to the surface of the fibers, thereby improving the roughness of the composite film during film formation, which further controls the hydrophobicity and optical properties. Through observation of the 0.5 wt% of HS_n_D_6_ and D_6_ + HS_n_ suspensions and films, the transparency of the composite films decreased with increasing HN-SiO_2_ content, and the composite suspensions prepared by one-step ball milling had higher transparency.

In the one-step preparation process of hydrophobic composite, although the weight of initial cellulose and HN-SiO_2_ is known, the content of HN-SiO_2_ may change due to operations such as modification and centrifugation in the process. To estimate the actual HN-SiO_2_ content in the HS_n_D_6_, thermogravimetric analysis is used to measure the HN-SiO_2_ content, which is the preferred choice with very small errors, especially for small sample amounts [36]. The actual weight of HN-SiO_2_ is calculated by Equation (3).

(3) $$Actual\;weight\;of\;HN-SiO_{2}\left(\%\right)=\frac{Final\;weight}{89.73}\times 100\%$$

As shown in Table 1 and Figure S1, the actual proportion values of HN-SiO_2_ are close to the theoretical values, and the deviations are mainly positive, which may be due to the high residual caused by the flame-retardation effect of silica particles. As mentioned in the literatures, the addition of nano-silica inhibits the decomposition of cellulose at high temperature to produce carbon dioxide, water vapor, and other gases, and promotes cellulose carbonization [37,38,39]. These factors make the final residual weight higher, which is consistent with the experimental results. Overall, the samples prepared by the two different methods are comparable in subsequent tests.

### 3.1. Chemical Structure of CNF in HS_n_D_6_

**Figure 2:**
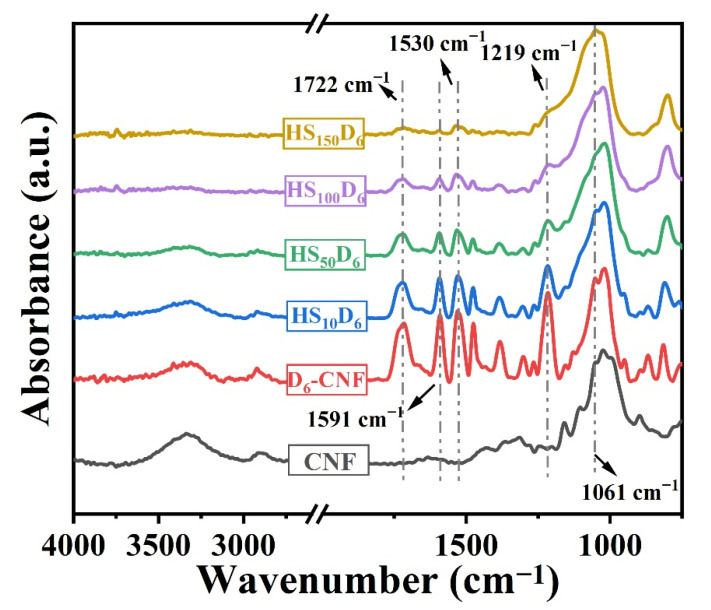
FTIR spectrum of CNF and HS_n_D_6_ films.

In order to prove that DCPI and cellulose can still undergo urethane reaction in the presence of HN-SiO_2_, the FTIR spectra of HS_n_D_6_ with different nanoparticle contents were tested (Figure 2). Compared with modified D_6_-CNF, the increase of HN-SiO_2_ content leads to a decrease in the intensity of the -OH stretching vibration peak at 3414 cm^−1^. The chemical structure changes of the modified CNF can still be observed from FTIR spectra. The peaks at 1722 cm^−1^ correspond to the stretching of C=O of carbamate. Absorption frequencies of carbamate bond appear at 1530 cm^−1^. The peaks at 1591 cm^−1^ and 1219 cm^−1^ correspond to the skeleton vibration and =C-H in-plane bending of benzene ring, respectively. Therefore, the presence of HN-SiO_2_ does not affect the chemical reaction between cellulose and DCPI.

### 3.2. Morphology of HS_n_D_6_

**Figure 3:**
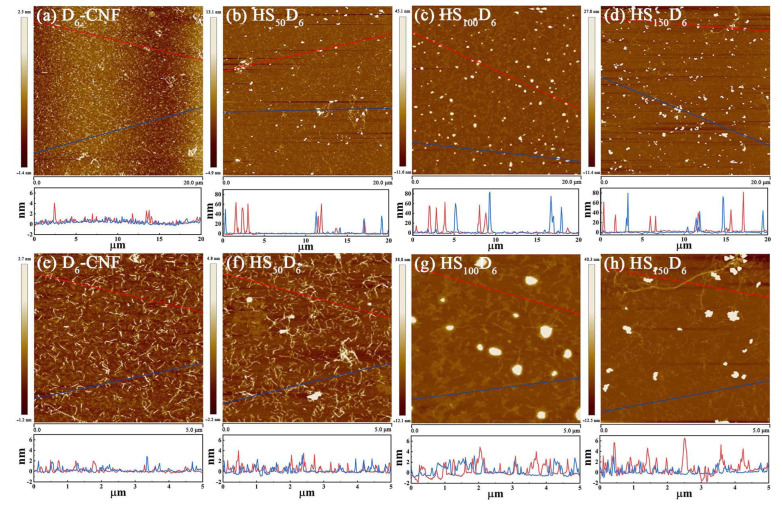
The AFM images of morphology of (**a**) D_6_-CNF and HN-SiO_2_ in (**b**) HS_50_D_6_, (**c**) HS_100_D_6_, and (**d**) HS_150_D_6_. The AFM images of CNF in (**e**) D_6_-CNF, (**f**) HS_50_D_6_, (**g**) HS_100_D_6_, and (**h**) HS_150_D_6_.

The morphology of HN-SiO_2_ and CNF in HS_n_D_6_ with different HN-SiO_2_ content was exhibited in Figure 3 and Figure S2 (the raw date). The size of HN-SiO_2_ clusters was smaller than 90 nm, which benefited from the weakened agglomeration behavior of HN-SiO_2_ by the effect of CNF and ball milling. Moreover, such nanoparticle clusters with different sizes are also beneficial to construct micro- and nano-scale rough structures. In order to further observe the effect of silica on the defibrillation of cellulose, we enlarged Figure 3a–d to measure the diameter of CNF. It is worth noting that when the dispersion concentration was 5 ppm, CNF formed a film on the surface of the mica sheet (Figure 3e). The fibers exposed on the film were entirely nanofibers less than 3 nm in diameter with the simultaneous cutting of the fibrils into short micrometers long segments. With the increase of HN-SiO_2_ content, the diameter of CNF increased by 2–6 nm, while the degree of defibration of cellulose will not be affected.

### 3.3. Morphology of CNF/HN-SiO_2_ Films

**Figure 4:**
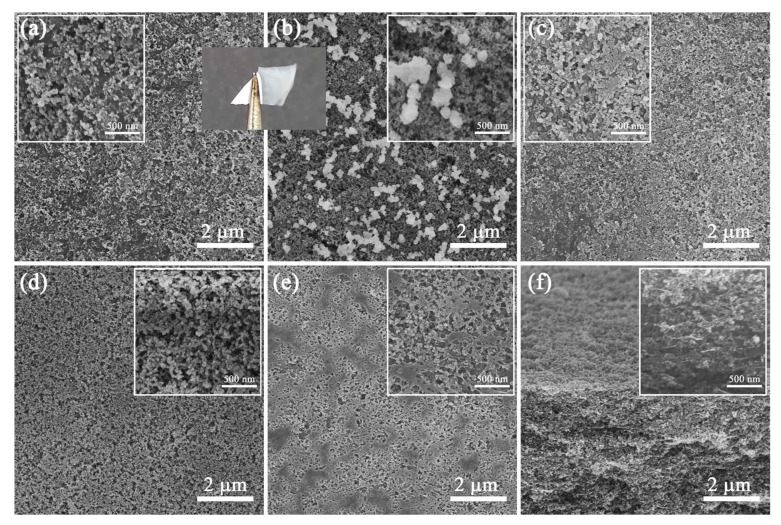
SEM images of D_6_ + HS_150_ films (**a**) before and (**b**) after combustion. SEM images of HS_150_D_6_ films (**c**) before and (**d**) after combustion. SEM images of (**e**) bottom-section and (**f**) cross-section of HS_150_D_6_ films.

To explore the effect of ultrasonic and ball-milling methods on the interaction between nanoparticles and CNF, the film morphologies of HS_150_D_6_ and D_6_ + HS_150_ were characterized (Figure S3 is the raw date). As shown in Figure 4a–d, the HN-SiO_2_ clusters in D_6_ + HS_150_ were larger than those in HS_150_D_6_, which indicated that the effect of ball milling and CNF could hinder the agglomeration behavior of HN-SiO_2_. This is because with the formation of CNF, HN-SiO_2_ can be further dispersed on the CNF surface by ball milling. Figure 4e shows the CNF/HN-SiO_2_ film close to the glass, i.e., the morphology of the film formed at the interface. Figure 4f shows the cross-sectional HN-SiO_2_ distribution in composite films. It can be seen that the CNF uniformly binds the nanoparticles together during film formation, showing a situation of stacking layer by layer. When the composite forms a film, the CNF first tends to form a film at the interface and then adhere the nanoparticles to the surface. Therefore, the modification and defibrillation of cellulose by ball milling will further promote the dispersion of nanoparticles, which is advantageous compared with conventional dispersion methods.

### 3.4. Wetting Properties of CNF/HN-SiO_2_ Films

**Figure 5:**
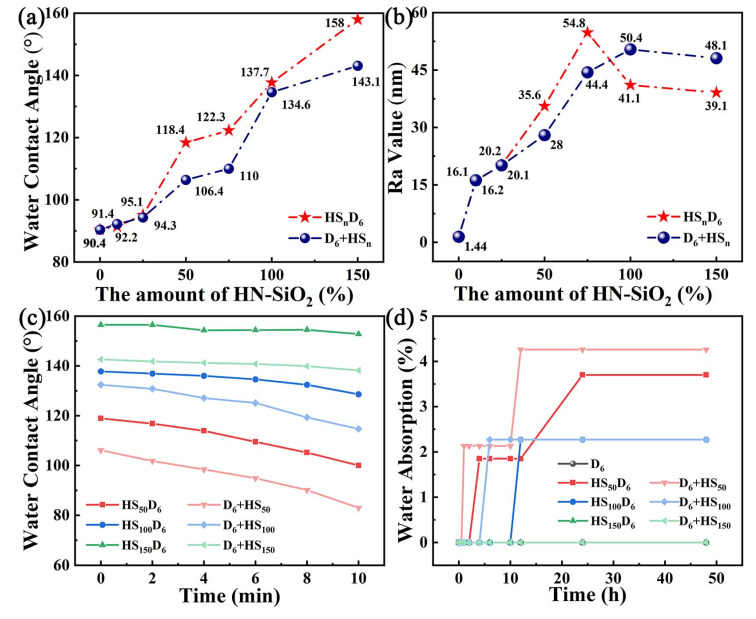
(**a**) Water contact angle and (**b**) Ra value of CNF/HN-SiO_2_ films. (**c**) Change of WCA and (**d**) hygroscopicity of HS_n_D_6_-film and D_6_ + HS_n_-film with different time.

To understand the influence of HN-SiO_2_ content and dispersion method on the hydrophobicity property, the HS_n_D_6_ and D_6_ + HS_n_ composite films with various weight ratios of HN-SiO_2_ to CNF ranging from 0 to 1.5 were investigated and the results are presented in Figure 5. As shown in Figure 5a, with a low HN-SiO_2_ content, low WCA was observed while the large content exhibited better hydrophobicity because the hydrophobicity of HN-SiO_2_ is better than modified CNF. When the weight ratio of HN-SiO_2_ to CNF was less than 0.75, the WCA and roughness increased with increasing HN-SiO_2_ content, whether it was HS_n_D_6_ or D_6_ + HS_n_ films, which was also in line with the formation of rough structure on the film surface by HN-SiO_2_. However, after the weight ratio was higher than 0.75, increasing HN-SiO_2_ content led to an increase in WCA, while the surface roughness of the HS_n_D_6_ film decreased, which implied that HN-SiO_2_ particles were more evenly distributed under the mechanical forces of ball milling. Meanwhile, it was noticed that the HS_n_D_6_ film became super-hydrophobicity with the WCA of 158.0° when the weight ratio was 1.5, while D_6_ + HS_n_ only reached 143.1° at the same dosage and the Ra value of the HS_n_D_6_ film is smaller than that of the D_6_ + HS_n_ film. Combining with Figure 4a–d, it can be seen that the micro/nano structure of the nanocomposite coating prepared by the one-step ball milling method is more similar to the dense layer of lotus leaf structure [4], which further endows better hydrophobic properties. As shown in Figure 5c–d, the hydrophobicity and water absorption of HS_n_D_6_-film is better than that of D_6_ + HS_n_-film, which can be concluded from the variation trend of WCA with different residence time. Moreover, the stability of composite films is improved with the increase of hydrophobic SiO_2_ content. Therefore, it is advantageous to complete the dispersion of nanoparticles by ball milling.

The present reported hydrophobic materials by mixing hydrophobic SiO_2_ and cellulose-based materials in recent years are listed in Table 2. Benefitting from the cellulose-based mechanochemical modification dispersion method, the HS_150_D_6_ coating exhibits high super-hydrophobicity with a water contact angle over 158°.

### 3.5. Optical Properties of CNF/HN-SiO_2_ Films

**Figure 6:**
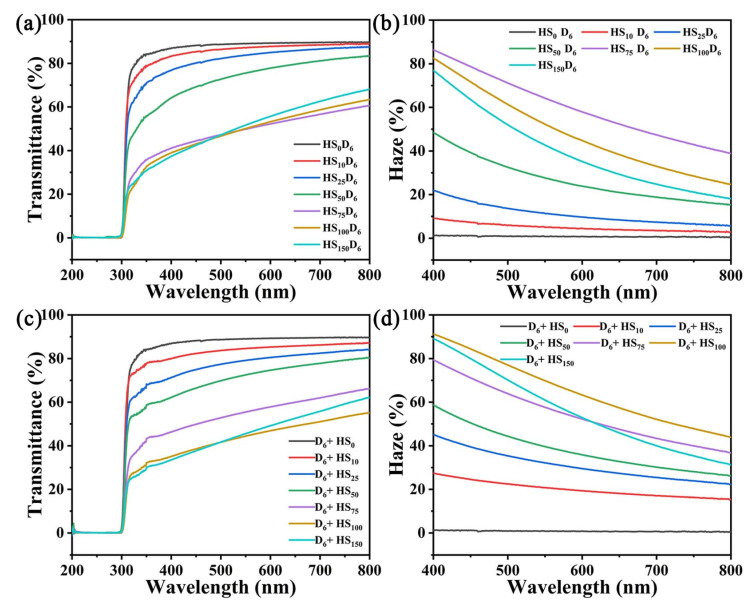
(**a**) Transmittance and (**b**) haze of HS_n_D_6_ films. (**c**) Transmittance and (**d**) haze of D_6_ + HS_n_ films.

The changes in transparency and haze of HS_n_D_6_ and D_6_-HS_n_ films were shown in Figure 6. The transmittance and haze values of D_6_-CNF film at 550 nm were 90% and 1%, respectively. With the increase of HN-SiO_2_ content, the transparency of CNF/HN-SiO_2_ composite films first decreased and then increased, accompanied by the opposite change of haze value. When the weight ratio of HN-SiO_2_ to CNF was less than 0.50, the transmittance of the composite films at 550 nm remained above 80%. The transmittance of the HS_n_D_6_ film reached the minimum value of 50%, and the haze value reached maximum value of 64% when the weight ratio is 0.75. The difference is that when the weight ratio is 1, the transmittance of the D_6_ + HS_n_ film reached the minimum value of 44%, and the haze reached the maximum value of 70%. The results further show that the dispersion uniformity of ball milling is better. The optical properties of the film are mainly affected by light scattering and the changes in roughness are consistent with changes in optical properties [46]. Therefore, the roughness of the chemically hydrophobic surface is further increased by controlling the content of HN-SiO_2_, thereby regulating the hydrophobic properties and optical properties of the film.

### 3.6. Wetting Properties of HS_n_D_6_ Coated Paper

**Figure 7:**
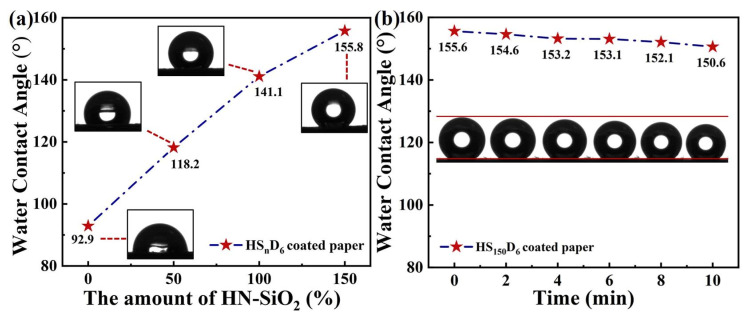
(**a**) Water contact angle of HS_n_D_6_ coated paper. (**b**) Change of WCA of HS_150_D_6_ coated paper.

**Figure 8:**
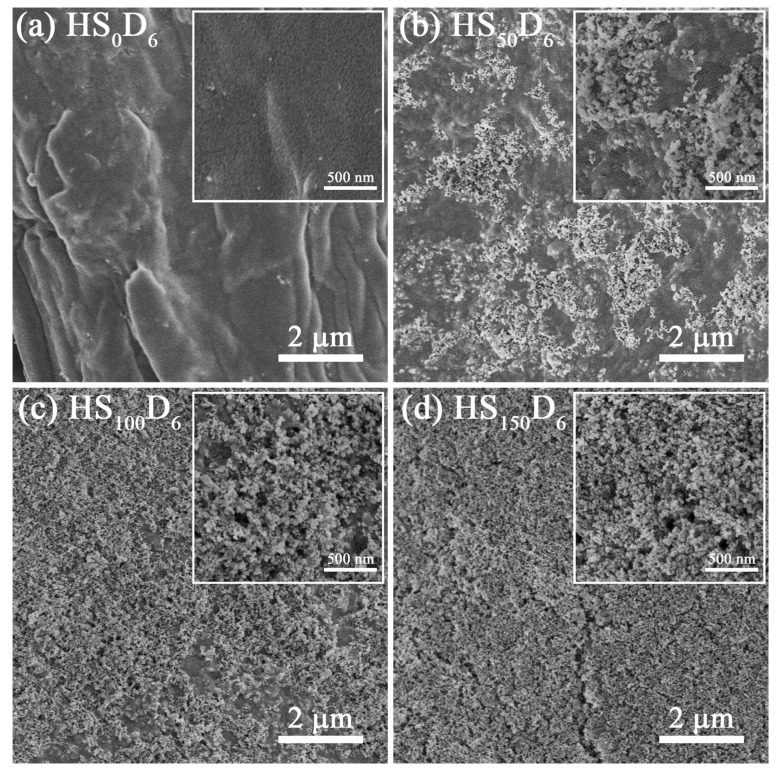
The SEM images of (**a**) HS_0_D_6_, (**b**) HS_50_D_6_, (**c**) HS_100_D_6_, and (**d**) HS_150_D_6_ coated paper.

The application of HS_n_D_6_ dispersion as a hydrophobic coating on paper was also investigated (Figure 7). The WCA of HS_n_D_6_ coated paper increased with increasing HN-SiO_2_ content, and it can also impart super-hydrophobicity to the paper surface at the weight ratio of HN-SiO_2_ to CNF is 1.5. Figure 8 and Figure S4 (the raw date) shows the variation of the surface morphology of the coated paper with different HN-SiO_2_ content. When the kraft paper is modified only by D_6_-CNF, it can be clearly observed that the fibers on paper surface are covered by dense D_6_-CNF (Figure 8a), thereby imparting paper hydrophobicity. As the HN-SiO_2_ content increasing, the nanoparticles exposed on the paper surface increased to construct micro/nano structures, in accordance with the theory of Cassie model. It is noted that the WCA did not change much on coated paper but the volume of water droplets was shrinking when the weight ratio is 1.5. This is because the treated surface is water repellent, whereas the untreated side of the paper is hydrophilic, resulting in Janus-type wetting and wicking properties [47] (Figure 7b). The modified CNF in the coating tends to form a film on paper surface, further prompting the exposure of HN-SiO_2_ to construct micro/nano structures on CNF-film, which leads to the super-hydrophobicity on the paper surface, while the other side of the paper maintains its original hydrophilicity. Because of the Janus properties, the coated paper can act as water transport and storage devices.

## 4. Conclusions

In this work, a facile mechanochemical method was adapted to prepare hydrophobic CNF/HN-SiO_2_ mixture suspensions. Compared with ultrasonic dispersion, ball milling can obtain better dispersed composite coatings with the average particle size of HN-SiO_2_ clusters less than 90 nm. The resultant CNF/HN-SiO_2_ product showed good film-forming, hydrophobic properties, and tunable optical properties by changing the content of HN-SiO_2_. When the weight ratio of HN-SiO_2_ to CNF is 1.5, a uniform film can still be obtained with the WCA of 158° and its coated paper also acquires super-hydrophobicity with WCA of 155.8°. Therefore, such nanoparticle dispersion method and CNF-based hydrophobic coatings are expected to be a potential route for fabricating various cellulose-based multifunctional materials.

## Figures and Tables

**Table 1:** Determination weight percent of HN-SiO_2_ in HS_n_D_6_ by TGA.

Sample	Final Weight (%)	Theoretical Weight of HN-SiO_2_ (%)	Actual Weight of HN-SiO_2_ (%)	Deviation (%)
D_6_-CNF	0	0	-	-
HN-SiO_2_	89.73	100	-	-
HS_10_D_6_	13.21	9.1	14.7	5.6
HS_50_D_6_	30.35	33.3	33.8	0.5
HS_100_D_6_	48.40	50.0	53.9	3.9
HS_150_D_6_	58.81	60.0	65.5	5.5

**Table 2:** Comparison of hydrophobic SiO_2_ and cellulose-based composite in recent literatures.

Year	Materials	Method	WCA (°)	Ref
2013	aerogels	the freeze-drying and cold plasma modification technology.	132	[40]
2014	aerogels	the sol–gel process and the supercritical drying method by DMSO	138	[41]
2015	aerogels	the sol-gel and freeze-drying method.	146	[42]
2016	film	one-pot method	137	[20]
2018	film	a two-step method involving a SiO_2_ sol-gel process	121	[43]
2021	coated fabric	double coated construction by a simple multi-step dipping	156.6	[44]
2022	coated paper	the laminated process followed by spraying approach	151.2	[45]
this work	film and coated paper	one-step mechanochemical by ball milling	158	-

## Data Availability

The data presented are contained within the article.

## Supplementary Materials

The following supporting information can be downloaded at: https://www.mdpi.com/article/10.3390/polym14204413/s1, Figure S1: TGA of D6-CNF, HN-SiO_2_, and HSnD6 (Table 1). Figure S2: The raw data of Figure 3; Figure S3: The raw data of Figure 4; Figure S4: The raw data of Figure 8.
