# Supplementary material for: Preparing Hydrophobic Cellulose Nanofibers-SiO2 Films and Coating by One-Step Mechanochemical Method
Source: Polymers (Basel). 2022 Oct 19;14(20):4413. doi: 10.3390/polym14204413 (PMC9611666; doi:10.3390/polym14204413)
Supplement: Supplementary file 1 [file polymers-14-04413-s001.zip › polymers-1965649-supplementary.pdf]

polymers

MDPI

Supplementary Material: Preparing Hydrophobic Cellulose Nanofibers-SiO_2 Films and Coating by One-Step Mechanochemical Method

Xi Chen, Lijiaqi Zhang, Min Wu, and Yong Huang

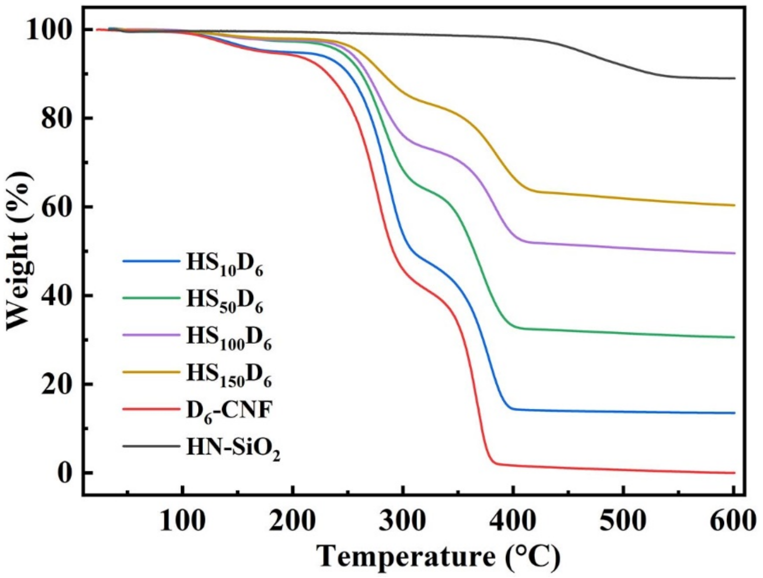

Figure S1. TGA of D_6-CNF, HN-SiO_2, and HS_nD_6.

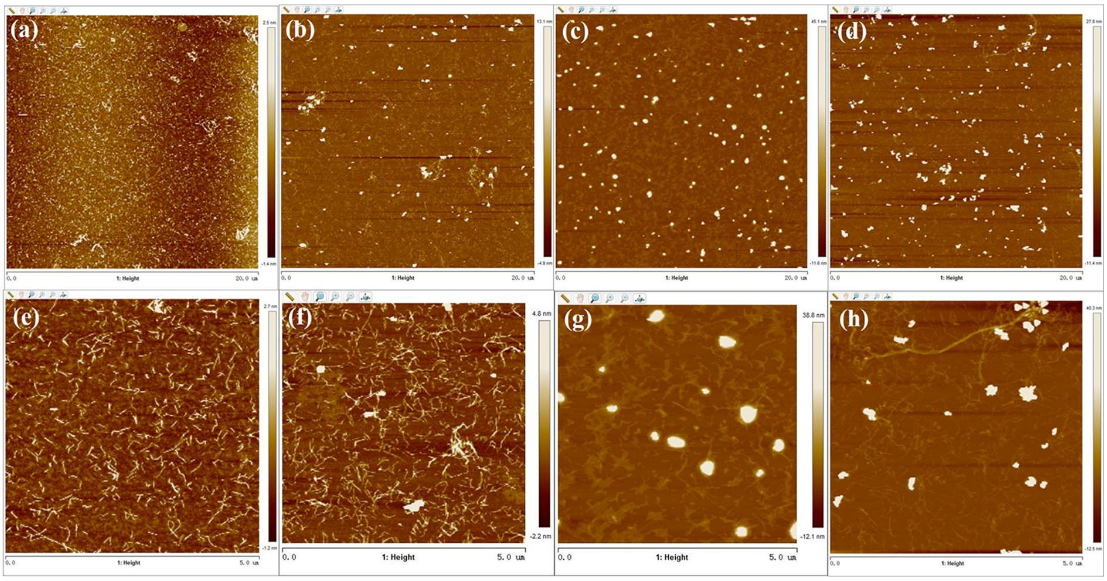

Figure S2. The AFM images of morphology of (a) D_6-CNF and HN-SiO_2 in (b) $HS_{50}D_6$, (c) $HS_{100}D_6$, (d) $HS_{150}D_6$. The AFM images of CNF in (e) D_6-CNF, (f) $HS_{50}D_6$, (g) $HS_{100}D_6$, (h) $HS_{150}D_6$.

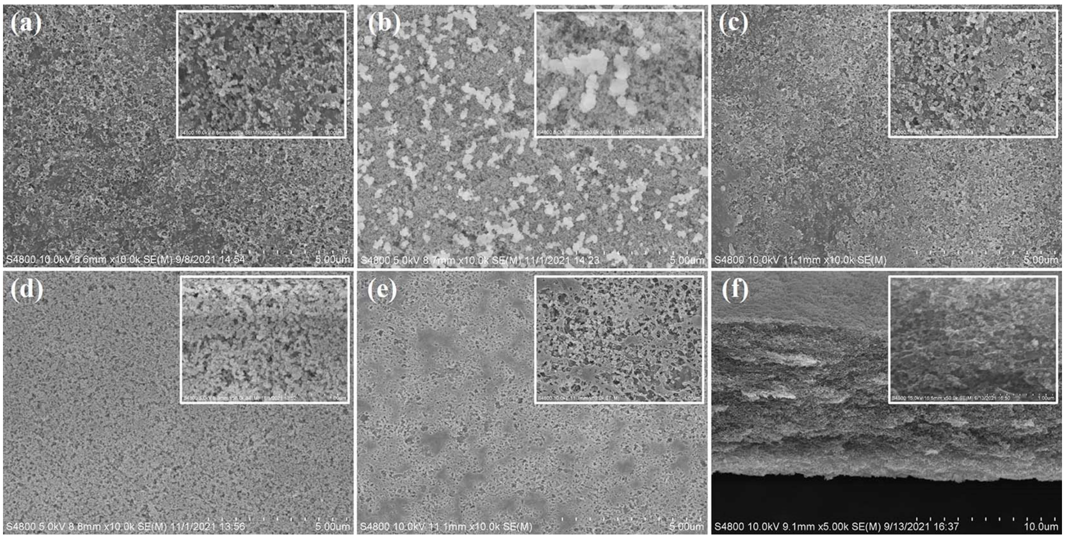

Figure S3. SEM images of (a) D_6+HS_{150} films before and (b) after combustion. (c) $HS_{150}D_6$ films before and (d) after combustion. (e) bottom-section and (f) cross-section of $HS_{150}D_6$ films.

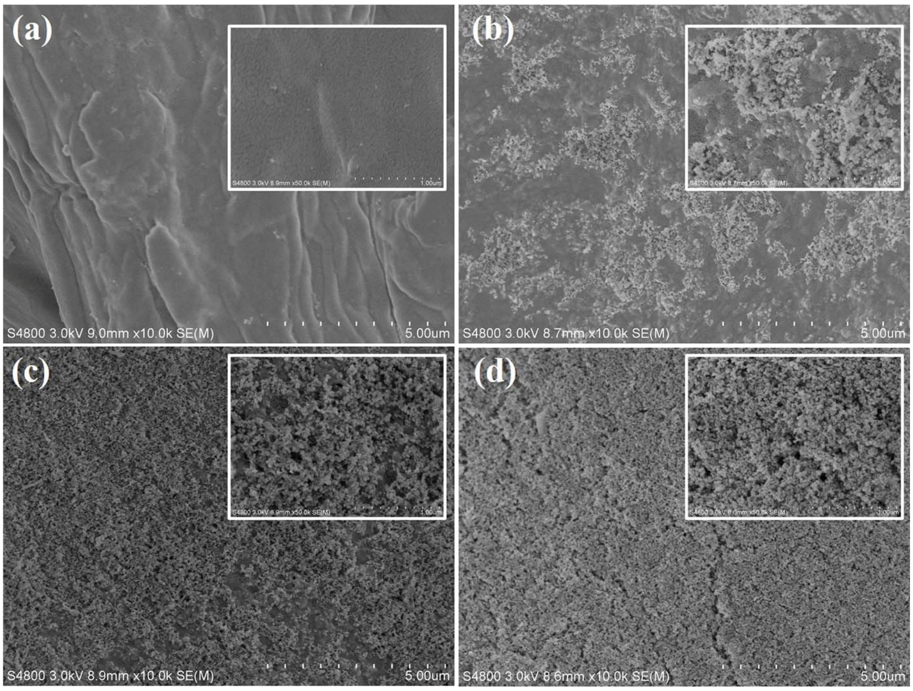

Figure S4. The SEM images of (a) D_6-CNF, (b) $HS_{50}D_6$, (c) $HS_{100}D_6$, (d) $HS_{150}D_6$ coated paper.
